# Evaluating the Prevalence of Leading Practices in Antimicrobial Stewardship

**DOI:** 10.1017/ash.2021.75

**Published:** 2021-07-29

**Authors:** Barbara Braun, Salome Chitavi, Eddie Stenehjem, Mushira Khan, David Baker, David Hyun

## Abstract

**Background:** Most hospitals have a basic infrastructure in place for antimicrobial stewardship programs (ASPs). Although this is a critical first step, we need to ensure that ASPs are working to implement effective evidence-based approaches nationally. In 2018, a group of leading antibiotic stewardship organizations met and identified specific, effective, and recommended ASP activities based on current scientific evidence and their experience (Baker et al, *Joint Comm J Qual Pat Saf* 2019;45:517–523). To determine the extent to which hospitals are currently implementing the recommended practices, we conducted an electronic questionnaire–based assessment. **Methods:** A 50-item questionnaire-based assessment was sent via QualtricsTM to the hospital’s designated ASP leader. The sample comprised 992 Joint Commission accredited hospitals. The practices of interest related to (1) development of facility-specific treatment guidelines, (2) measuring appropriate use and concordance of care with these guidelines, (3) engaging clinicians while the patient is on the unit, (4) diagnostic stewardship, (5) measurement of antimicrobial utilization data, and (6) measuring hospital-acquired *Clostridioides difficile* infection (CDI) rates. Sampling weights were used to adjust the results for nonresponse using R software. **Results:** In total, 288 hospitals completed the questionnaire. Small and nonteaching hospitals were significantly less likely to respond (p < 0.005, p=0.01 respectively), however there were no differences by healthcare system membership or urban/rural location. 49% of respondents had the specialist term ASP or infectious disease (ID) in their title. Most hospitals (93.1%) had developed facility-specific treatment guidelines for specific inpatient conditions, often community-acquired pneumonia (85%), sepsis (81%), UTI (75%), and SSTI (69%). However, only 37% had formally assessed compliance with 1 or more of these guidelines. Also, 83% reported having a process for prospective audit and feedback, of which 43% do this 4–5 days per week. Similarly, 49% reported that they review all antimicrobials ordered. Recommendations are commonly given by the ASP pharmacist (69%) via some combination of telephone (78%), face-to-face (69%), text message (54%), and/or EHR alert (36%). Overall, 66% of hospitals had procedures in place to prevent inappropriate diagnostic testing for *C. difficile*, and 39% of hospitals had similar policies for urine specimens. Furthermore, >80% were routinely measuring days of therapy and CDI rates. **Conclusions:** Most hospitals have facility-specific treatment guidelines and measure CDI and days of therapy. Practices for active engagement with frontline staff in prospective audit and feedback vary widely. Greater understanding of barriers to assessing adherence to hospitals’ treatment guidelines is critical to improving this practice.

**Funding:** The Pew Charitable Trusts

**Disclosures:** None

